# Editorial: Case reports in autoimmune and autoinflammatory disorders, volume II

**DOI:** 10.3389/fimmu.2026.1968426

**Published:** 2026-09-01

**Authors:** Chris Wincup, Gian Marco Ghiggeri

**Affiliations:** 1 Department of Clinical and Academic Rheumatology, King’s College Hospital, London, United Kingdom; 2 Centre for Rheumatic Diseases, King’s College London, London, United Kingdom; 3 Nephrology, Institute Giannina Gaslini, Genoa, Italy

**Keywords:** autoimmune encephalitis, autoimmunity, autoinflammation, case report, checkpoint inhibitor, immunotherapy, overlap syndrome, vasculitis

## Introduction

Autoimmune and autoinflammatory disorders remain among the most diagnostically and therapeutically challenging conditions in clinical medicine. They cross every organ boundary, present with high levels of both immunological and phenotypic heterogeneity, and are increasingly present against a background of potent immunomodulatory therapy that can itself provoke, unmask, or reshape immune-mediated disease. It is on this background that the atypical case is very often where the field’s assumptions are tested first. It is in the single, carefully documented patient that a novel autoantibody is first tied to a syndrome, that an off-label therapy is first seen to work or to fail, and that an unfamiliar toxicity of an established drug is first flagged (often years before any of these observations accumulate into the numbers required for systematic study).

Volume I of this Research Topic set out to demonstrate the value of the well-constructed case report in supplementing the wider literature, and the appetite for that format has, if anything, grown. Volume II comprises fifty-two contributions, drawn from centers across Europe, Asia, North America and the Caribbean, spanning neurology, nephrology, rheumatology, endocrinology, dermatology and haemato-oncology. The breadth of what has been submitted is a fair reflection of how porous the borders of this discipline have become, and of how much of its most clinically useful knowledge still enters the literature one patient at a time. Case reports carry well-recognized limitations, noting that they cannot establish causation, they are vulnerable to publication and reporting bias, and a single striking observation may not generalize. However, their value lies precisely in signaling early, and each of the reports that follows should be read in that spirit.

Early findings from case reports have arguably become more, not less, important in the biologic era. As the therapeutic armamentarium has shifted from broad immunosuppression toward agents defined by a single molecular target or cell population, the number of possible drug–disease pairings has expanded far faster than the capacity of randomized trials to test them, and much of the earliest evidence for using a targeted agent outside its licensed indication necessarily takes the form of the individual report. The same era has produced entirely new disease entities, including the immune-related adverse events of checkpoint inhibition and the secondary autoimmunity of lymphocyte-depleting therapy, which were first described one patient at a time. A well-documented case is therefore not merely a supplement to trial evidence but, for a substantial part of this field, the format in which new knowledge first becomes available at all.

Rather than catalogue these reports by organ alone, we have organized this Editorial around a structure that follows both where disease presents and how the field is moving. Two clusters are grounded in clinical territory (autoimmune neurology and systemic/organ-specific autoimmunity). Three further clusters cut across organ boundaries and capture the mechanistic and practical themes that recur throughout the Research Topic, including the rapid expansion of the targeted-therapy toolkit, the growing problem of iatrogenic and checkpoint-inhibitor-associated autoimmunity, and the perennial challenge of diagnostic complexity, overlap and mimicry. Many reports could sit comfortably in more than one grouping, which is itself the point.

## Autoimmune neurology and neuroimmunology

The single largest body of work in this volume concerns the nervous system, and it captures both the diagnostic precision that antibody-defined syndromes now permit and the therapeutic modulation they invite. Several reports address the antibody-negative or treatment-refractory patient in whom conventional treatment algorithms are insufficient to bring about control of symptoms. Zheng et al. describe rituximab in antibody-negative combined central and peripheral demyelination presenting with acute respiratory failure, a presentation that sits between recognized categories and forces a pragmatic therapeutic decision. Cao et al. report a COVID-19–associated, delayed-onset, MuSK-positive myasthenia gravis whose sole manifestation was respiratory failure, a reminder that neuromuscular autoimmunity can present without the classical fatigable weakness that usually prompts the diagnosis. Pan et al. add a focused account of unilateral ophthalmoplegia as the presenting feature of anti-GQ1b antibody syndrome, extending the recognized phenotypic range of that entity.

A recurring strength of the neurological submissions is the willingness to reach for newer biologic agents when established immunosuppression fails. Tan et al. contribute the only case series in the Research Topic, describing sequential neonatal Fc receptor (FcRn) blockade followed by B cell depletion in three patients with treatment-refractory relapsing autoimmune encephalitis. This acts as an explicit attempt to combine antibody clearance with a durable reduction in antibody production. Deng et al. and Watanabe et al. both explore FcRn antagonism with efgartigimod, the former in Guillain-Barré syndrome and the latter in refractory anti-GQ1b antibody syndrome coexisting with myasthenia gravis, together illustrating how a mechanism developed for one antibody-mediated disease is being tested rapidly across the antibody-mediated neurological spectrum. Lin et al. report telitacicept, a dual BAFF/APRIL inhibitor, in glial fibrillary acidic protein (GFAP) autoimmune astrocytopathy, and Chen et al. describe satralizumab in the rare co-occurrence of neuromyelitis optica spectrum disorder (NMOSD) with capillary leak syndrome. Beyond the antibody-defined syndromes, von Zedtwitz et al. examine acute polymorphic psychosis in the presence of serum NMDA-receptor IgG antibodies, a report that engages carefully with the difficult question of when a serum antibody is pathogenically relevant to a psychiatric presentation, and Bayraktar Eltutan et al. describe pediatric CNTN1 antibody-associated neuropathy with concurrent nephropathy, a nodo-paranodal syndrome whose renal involvement underscores that these antigens are not confined to the nervous system. What unites these otherwise disparate reports is a common clinical logic highlighting that as the target antigen is defined with ever greater precision, the phenotype attached to it widens rather than narrows, and treatment is increasingly chosen to match the presumed effector mechanism. The nervous system, where the antigens are best characterized, is where this logic is currently most visible, but it is not confined there.

## Systemic and organ-specific autoimmunity

The second major cluster gathers reports across the systemic autoimmune disease, the vasculitides, and organ-specific autoimmunity of the kidney, endocrine system and skin, where the recurring lesson is the unexpected site or severity of involvement. Systemic lupus erythematosus is well represented and, characteristically, rarely behaves conventionally. Huang et al. describe lupus mesenteric vasculitis masquerading as urticaria with abdominal pain, followed over ten months. Wang et al. report bilateral adrenal hemorrhage in a patient with lupus and antiphospholipid syndrome. Zhang et al. present neuropsychiatric lupus complicated by primary central nervous system diffuse large B cell lymphoma, a juxtaposition that raises difficult questions about the interplay of chronic immune activation and lymphomagenesis. Liu et al. add a pediatric account of Fabry disease overlapping with lupus, a metabolic-autoimmune overlap that is easily missed. Primary Sjögren’s disease likewise appears in unexpected guises with Tang et al. reporting simultaneous cerebral and coronary arterial involvement as the initial presentation, while Duan et al. describe membranoproliferative glomerulonephritis-like lesions within cryoglobulinemic glomerulonephritis.

The vasculitides and their mimics recur throughout the submissions to this article collection. Chenaf-Benabdelmoumene et al. describe late post-transplant recurrence of anti-myeloperoxidase (MPO) antibody-associated vasculitis in a former double-positive patient, a reminder that serological history matters long after apparent clinical quiescence. Chen et al. report eosinophilic granulomatosis with polyangiitis (EGPA) presenting first with cerebellar infarction in a child, and Ye et al. document massive lower gastrointestinal hemorrhage from rupture of a superior mesenteric artery branch in Behçet’s disease, both cases emphasize a life-threatening vascular event was the entry point to the diagnosis. Ouerdani et al. explore the boundary between urticarial hypocomplementemic vasculitis syndrome and lupus, a distinction with real prognostic and therapeutic consequence.

Renal autoimmunity forms a distinct thread within this section. Li et al. describe rapidly progressive anti-glomerular basement membrane (GBM) disease arising on a background of long-standing rheumatoid arthritis, and Zhu et al. report the concurrence of IgG4-related tubulointerstitial nephritis with renal AA amyloidosis, an instructive combination of two mechanistically distinct processes within the same individual. Adult-onset Still’s disease supplies two contrasting phenotypes with Zhang et al. expand its recognized renal spectrum with focal proliferative glomerulonephritis and ischemic nephropathy, while Gomes et al. highlight a flagellate cutaneous eruption of adult-onset Still’s disease. The muscle and lung are represented by Li et al., who report an acute exacerbation of anti-Ha antibody-positive antisynthetase syndrome-associated interstitial lung disease.

Endocrine and dermatological autoimmunity round out this cluster. Gao et al. and Hu et al. both address insulin autoimmune and exogenous insulin antibody syndromes, with the latter complicated by chronic kidney disease and long-standing type 2 diabetes. Su et al. describe familial type 2 diabetes coexisting with hypothyroidism and multiple autoimmune diseases, a cluster that speaks to shared autoimmune predisposition. In the skin, Li et al. report the coexistence of bullous pemphigoid, intrahepatic cholangiocarcinoma and alopecia areata as an example of multifactorial autoimmunity in a surgical context, and Chen et al. describe type A lymphomatoid papulosis initially presenting as a giant ulcer. A selection of pediatric and monogenic conditions also contribute here. Drago et al. report severe renal involvement in very early onset inflammatory bowel disease, and Zhang et al. describe X-linked lymphoproliferative disease presenting first with neurological symptoms. Across this heterogeneous cluster a single practical message recurs, the recognized organ footprint of a systemic autoimmune disease is consistently wider than its textbook description, and the presenting organ is frequently not the one the diagnosis would predict. Whether the entry point is the mesentery in lupus, the coronary and cerebral circulation in Sjögren’s, the adrenal gland, or the kidney in a disease usually defined by its serology, the report earns its place by documenting a site or a severity of involvement that shifts the index of suspicion for the next clinician who meets the same unfamiliar combination.

## An expanding targeted-therapy toolkit

If a single mechanistic theme runs through this volume, it is the speed with which mechanistically-defined biologic and small-molecule therapies are being deployed against diseases for which they were not originally licensed. The FcRn antagonists, BAFF/APRIL inhibition and targeting of interleukin-6 pathway already discussed in the neurological cluster are part of this same wave, and several further reports show it operating across other systems. The limits of mechanism-led enthusiasm are illustrated, unexpectedly, by Chalkia et al. who describe two patients with challenging manifestations of ANCA-associated vasculitis treated with the complement C5a-receptor antagonist avacopan. Their clinical observations stand, but the drug’s standing does not as since publication, the pivotal ADVOCATE trial has been retracted over data-integrity concerns. The episode is a salutary reminder that a mechanistically attractive, trial-endorsed therapy can still have its evidence base overturned. Yang et al. report dupilumab, an IL-4-receptor-α blocker, in Kimura disease, repurposing a type 2-directed therapy against an eosinophilic lymphoproliferative disorder. The Janus kinase inhibitors feature prominently with Chen et al. describing the response to two sequential JAK inhibitors over two years in a boy with STING-associated vasculopathy with onset in infancy (SAVI), a type I interferonopathy in which JAK blockade is mechanistically rational. Wang et al. report upadacitinib in refractory Behçet’s disease complicated by myelodysplastic syndrome with trisomy 8, offering mechanistic reflection on the hematological context. Application of B cell depleting is extended by Fu et al., who report rituximab for membranous nephropathy secondary to primary Sjögren’s disease. Read together (and alongside the FcRn antagonists, BAFF/APRIL inhibition and interleukin-6-receptor blockade deployed in the neurological reports above) these contributions trace a coherent mechanistic argument that runs through the entire volume. Where the effector pathway can be named, whether that is pathogenic IgG recycling, B cell help and antibody production, complement-mediated tissue injury, or interferon- and cytokine-driven inflammation, a therapy directed at that pathway is now being tried across whatever disease expresses it, and the licensed indication is treated as a starting point rather than a boundary. This is a rational and, in refractory disease, often the only available strategy, but it carries an evidentiary hazard that case reports must not obscure. A plausible mechanism and a temporally associated response are not the same as demonstrated efficacy, and the reports most useful to the field are those that describe the immunological rationale, the response and its durability, and any failures with equal candor. It is precisely this early, mechanism-anchored signal that is generated while formal trial evidence remains absent, which the case report is uniquely placed to record.

## Iatrogenic and checkpoint-inhibitor-associated autoimmunity

A distinct and growing category concerns autoimmunity that is provoked by treatment rather than arising *de novo*, a problem magnified by the expansion of immune checkpoint inhibition in oncology. Lee et al. report generalized lipodystrophy following checkpoint-inhibitor therapy, an unusual metabolic immune-related adverse event, and Anthon et al. describe malignant hypertension with multiorgan dysfunction during immunotherapy for gallbladder cancer. Other iatrogenic mechanisms are well represented. Teliti et al. discuss the management of alemtuzumab-induced Graves’ disease in pregnancy, a secondary autoimmunity arising after lymphocyte reconstitution and complicated by the additional considerations of gestation. Immunosuppression may also open the door to opportunistic infection that mimics or complicates the underlying disease. As Campolo et al. report parvovirus B19 encephalitis following rituximab in immune-mediated thrombocytopenia, and Chen et al. describe facial *Malassezia folliculitis* after infliximab in Crohn’s disease. Collectively these reports emphasize a clinical reality of modern practice, the therapies that control immune-mediated disease are themselves an expanding cause of it.

## Diagnostic complexity, overlap and mimicry

The final cluster gathers the reports whose central lesson is diagnostic. The overlap syndrome, the dual-antibody patient, and the mimic that misleads. Coexisting neuronal antibodies feature repeatedly as Liu et al. describe adolescent autoimmune encephalitis with dual anti-Drebrin and anti-mGluR2 antibodies. Zhang et al. report the coexistence of anti-LGI1 and anti-mGluR2 antibodies, and Zhu et al. present neuronal intranuclear inclusion disease coinciding with acute anti-CASPR2 encephalitis at the neuro-immune interface. Overlap across disease categories is equally instructive. Wang et al. report anti-aquaporin-4-positive NMOSD overlapping with “mixed connective tissue disease”, and Zhang et al. describe anti-GABA-B receptor limbic encephalitis arising in relapsing polychondritis, each pairing a neurological and a systemic autoimmune diagnosis. The problem of the convincing mimic is drawn out by Liu et al., who used metagenomic next-generation sequencing to identify *Rickettsia felis* infection in a patient with ankylosing spondylitis, and by Wang et al., whose patient was initially misdiagnosed with spinal infection rather than SAPHO syndrome. The are both cautionary accounts of the cost of anchoring diagnosis prematurely. Multi-system diagnostic challenges are captured by Tian et al., who report diabetic ketoacidosis with negative diabetes autoantibodies complicated by Guillain-Barré syndrome and reversible splenial lesion syndrome. This highlights three processes in one patient, none of which fully explains the others. The interface with malignancy recurs here too, most strikingly in Cheng et al.‘s account of unicentric Castleman disease with concurrent myasthenia gravis and paraneoplastic pemphigus. Finally, Tolmer et al. return attention to the laboratory itself, describing two atypical cases of an M6-like anti-mitochondrial antibody pattern unlinked to iproniazid, a reminder that the immunological assays on which these diagnoses rest continue to throw up patterns that demand careful interpretation.

## Concluding remarks

Taken as a whole, Volume II reinforces the argument that Volume I began. Fifty-two case reports, drawn from centres across the world and spanning almost every organ system, converge on a number of messages that matter directly for practice, that antibody-defined syndromes are widening the diagnostic vocabulary faster than trials can keep pace. This further emphasizes that mechanistically-targeted therapies are being carried rapidly from their original indications into rare and refractory disease. Furthermore, the treatments controlling immune-mediated disease are themselves a growing cause of it and overlap, mimicry and diagnostic anchoring remain the enduring hazards of this field. None of these lessons would be visible in aggregate data alone. The case report retains a specific and irreplaceable role at the leading edge of clinical immunology, whether by recording the first signal, describing the exceptional response, or flagging the adverse event before it is common enough to be systematically studied. We are grateful to every author who contributed to this Reseasrch Topic and to the reviewers whose scrutiny sustained its quality, and we look forward to further submissions that continues to give the atypical patient the careful attention that so often turns out to be instructive for all.

